# Large Unsaturated Magnetoresistance in Gated MoS_2_ Flakes

**DOI:** 10.1002/smll.202514561

**Published:** 2026-02-11

**Authors:** Anoir Hamdi, Dominik Dettmann, Andrés Rafael Botello‐Méndez, Atiye Pezeshki, Lilian Skokan, Andreas Ruediger, Gianluca Fiori, Zeila Zanolli, Emanuele Orgiu

**Affiliations:** ^1^ Centre Énergie Matériaux Télécommunications Institut national de la recherche scientifique Varennes Quebec Canada; ^2^ Chemistry Department Debye Institute for Nanomaterials Science and ETSF Condensed Matter and Interfaces Utrecht University Utrecht Netherlands; ^3^ Dipartimento di Ingegneria dell'Informazione University of Pisa Pisa Italy

**Keywords:** density functional theory transport simulation, field‐effect transistor, magnetotransport, transition metal dichalcogenides, van der Waals materials

## Abstract

Transition metal dichalcogenides (TMD) are a fertile playground to study the interactions between charge carriers and external magnetic fields. Van der Waals interlayer interactions enable the investigation of magnetotransport as a function of the different numbers of TMD layers. Here, we demonstrate unsaturated large magnetoresistance (MR) in MoS_2_ crystals by using a field‐effect transistor geometry to tune charge carrier density. Our work shows that the device can be operated in a given regime that allows reaching a maximum MR of 680% at 1.8 K without any sign of saturation. The device exhibits a higher sensitivity to magnetic fields when operating in the subthreshold regime than in its on‐state. Our work suggests that this effect stems from a change in the energy of the conducting states as observed by monitoring the threshold voltage shift. Notably, the magnitude of such a shift strongly decreases with temperature and number of layers. By means of Density Functional Theory calculations, we confirm that the origin of such a large MR is not the Lorentz force affecting band‐like transport, but rather, an interaction that affects the electronic properties of mid‐gap states in MoS_2_ that are dominating the charge transport at low temperature. This work demonstrates that controlling the charge density in the channel and the transport mechanism leads to engineering of the magnetic sensitivity in 2D materials.

## Graphical abstract

Magnetotransport investigation of layered MoS_2_ crystals in field‐effect devices reveals strong dependence on thickness and carrier density. Monolayer MoS_2_ exhibits the highest magnetoresistance of 680%. A combination of density functional theory and Boltzmann transport equation simulations shows that band transport is not causing the magnetic behavior, but rather defect‐mediated transport mechanisms enable the high magnetoresistance.

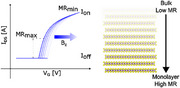

## 1 Introduction

2D materials represent a unique playground for emerging quantum phenomena such as exotic band structures [1], electron correlation [2], tunable excitons [3], and topological insulators [4, 5]. In particular, transition metal dichalcogenides (TMDs) attract significant interest due to their widely tunable properties [6, 7, 8, 9]. The choice of transition metal atom, chalcogen atom, layer number, and stacking symmetry provides a unique toolbox to synthesize a vast range of materials, including metal phases and semiconducting phases with tunable band gaps [10, 11]. Moreover, in comparison to graphene, the heavy metal atoms introduce strong spin‐orbit coupling to the system, lifting the degeneracy of the bands [12, 13]. The combination of the non‐degenerate bands and alternating broken inversion symmetry between odd and even numbers of layers of the 2H phase enables access to valley‐locked states [14]. Owing to their strong spin–orbit coupling, layer‐dependent symmetry, tunable carrier dynamics, and the emergence of topological and quantum transport effects, TMDs offer a versatile platform for exploring a wide range of magnetoelectric phenomena.

In particular, the effect of external magnetic fields on the band structure and charge transport attracts significant interest [15]. The latter one, often referred to as magnetoresistance (MR), is technologically relevant for applications such as sensing or magnetic memory storage [16]. MR is defined as $MR\;\left(B\right)=\frac{\rho (B)-\rho (0)}{\rho (0)}=\frac{R(B)-R(0)}{R(0)}$, where *ρ*(*B*) and *ρ*(*0*) are the resistivities and R(B) and R(0) are the resistances in the presence and absence of a magnetic field (B), respectively. It is generally believed that such an effect arises in a non‐magnetic material when the carrier movement is deflected by the external magnetic field through the Lorentz force [17]. In particular, when B is perpendicular to the current plane, the change in the material's resistivity is referred to as transverse MR. For applications, a high sensitivity is desired. However, a desired giant magnetoresistance (GMR) is difficult to obtain [18]. The saturation of MR is a major challenge, which limits the attainable sensitivity and requires novel approaches [19, 20]. In this regard, TMDs offer a plethora of possibilities to fine‐tune charge transport. Due to these properties, TMDs are an excellent material candidate for Hall sensors [21, 22, 23].

Henceforth, turning knobs to manipulate and engineer MR in these materials is highly sought after. One possible mechanism is the dynamic control of the Fermi level, which can significantly alter the MR properties [24]. Indeed, new charge transport regimes can be explored, such as the charge compensation mechanism, in which holes and electron contributions balance out [25, 26]. To the best of our knowledge, only a few studies have focused on devices with carrier density control [27, 28]. By exploiting the field effect, a tunable control over the Fermi level can be obtained, which enables the study of the magnetoresistance at different carrier density levels. MoS_2_ is a model system to study the effect of different carrier densities due to its strong spin‐orbit coupling and resulting non‐degenerate valence and conduction bands [12]. Moreover, the number of layers has a significant influence over the band structure, which causes the monolayer to exhibit a direct band gap and any thicker composite to be indirect [29]. In addition, MoS_2_ is an exceptionally strong candidate for the development of novel field effect transistor technologies owing to its high mobility and I_on_/I_off_ ratio [30, 31, 32, 33, 34]. By adding the tunability of the Fermi level to the magneto‐transport, the effect of the magnetic field on charge transport in carrier‐rich and low regimes can be explored. Moreover, TMDs exhibit a unique blend of different temperature‐dependent charge transport mechanisms [35]. At room temperature, band‐like transport governs the conduction mechanism. However, when T <200 K, the conduction in MoS_2_ starts to be dominated by a hopping mechanism and, at cryogenic temperature (<10 K), tunneling processes dominate the charge transport [22, 36, 37]. The incorporation of multiple transport mechanisms in the same 2D material provides an ideal platform to study the effect of magnetic fields on different transport regimes and identify the optimal conditions for high MR.

Here, we demonstrate a non‐saturated magnetoresistance in monolayer, few‐layer, and bulk MoS_2_ FET devices. The magnetotransport was found to be strongly tunable by means of the gate field. The devices exhibit relatively low magnetoresistance when the transistor is operated in the ON‐state. However, gradually turning the device into the threshold regime gives rise to a rapid increase in the magnetoresistance. A maximum value of 680% MR is obtained for a MoS_2_ monolayer at 1.8 K and 9 T with no sign of saturation. The absence of saturation is also observed in few‐layer and bulk devices. Our measurements demonstrate that the position of the Fermi level and the resulting increase in charge carrier density in the channel strongly influence the transport properties in the presence of a magnetic field. To reveal the underpinning mechanism of this modulation, we have calculated the magnetotransport properties of MoS_2_ using Density Functional Theory to solve the Boltzmann transport equation. We show that the experimentally measured MR trend cannot be reproduced if one only assumes an acting Lorentz force within electron‐phonon‐limited band transport. Furthermore, the high MR seems to depend on the manipulation of the energy levels and their effect on the charge transport, as evidenced by a shift in threshold voltage. The strong temperature dependence of this shift follows the reduction of MR at higher temperatures, demonstrating a profound effect of the magnetic field on the charge carriers. Our work demonstrates the potential of achieving significantly higher MR by preparing suitable device conditions and exploring the intriguing transport physics in devices by studying MR in different charge carrier regimes.

## 2 Results and Discussion

**FIGURE 1:**
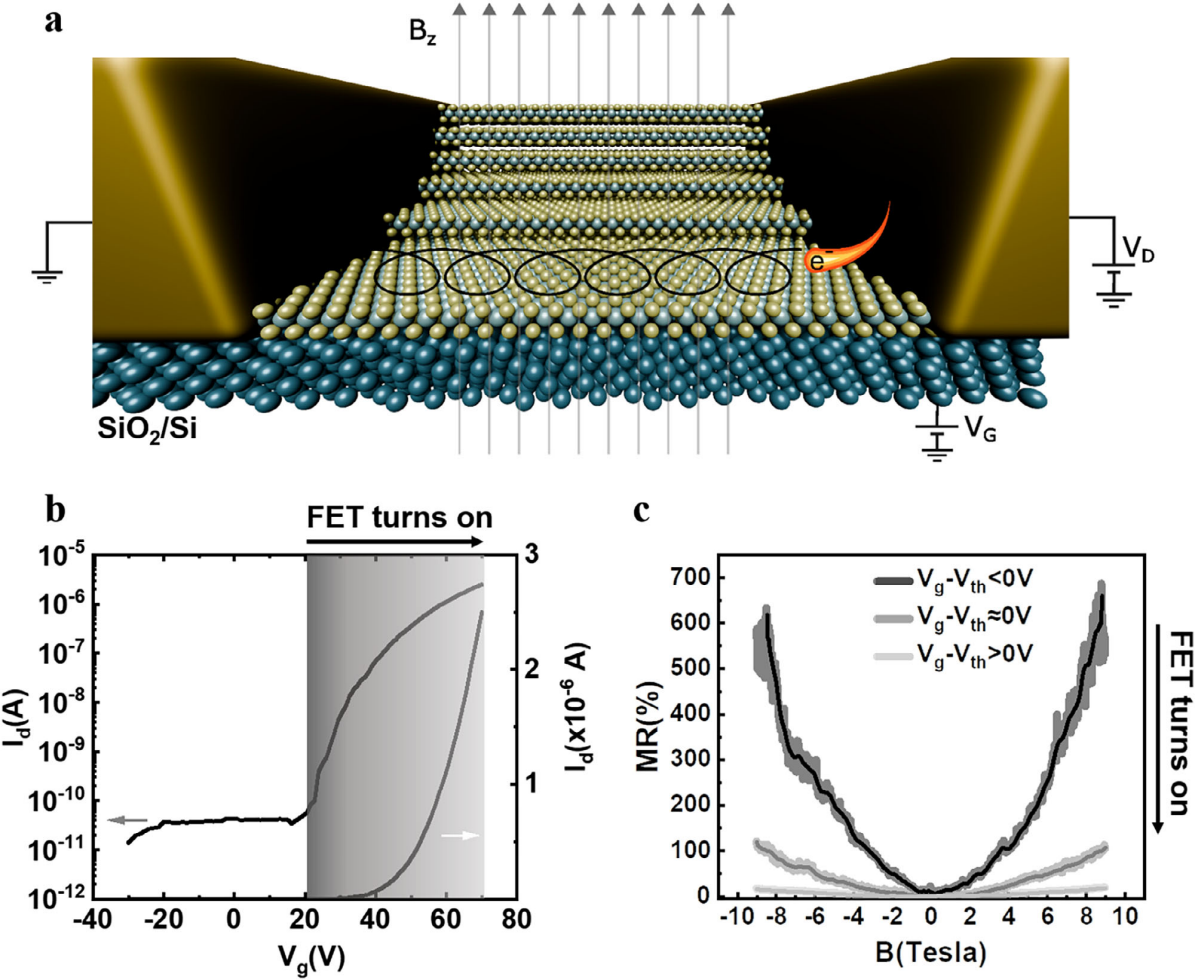
Magnetotransport in MoS_2_ FET devices. (a) Schematic of the device geometry employed in this work. (b) Transfer characteristics of monolayer MoS_2_ field‐effect transistors, without any applied magnetic field, plotted in both linear and logarithmic scales [V_d_ = 3 V, L typically ranges between 4 µm and 6 µm, W = 10 µm, t*_SiO2_* = 285 nm, T = 1.8 K]. (c) Magnetoresistance in a monolayer MoS_2_‐FET measured at 1.8 K in different transistor operation regimes: subthreshold region (V_g_ –V_th_ <0 V), above threshold region (V_g_ –V_th_ > 0), and at the threshold region (V_g_ –V_th_ = 0 V). [L ranges typically between 4 µm and 6 µm, W = 10 µm, t*_SiO2_* = 285 nm, T = 1.8 K].

We fabricated transistors having either monolayer (ML), few‐layer or bulk MoS_2_ as the semiconducting layer by mechanical exfoliation (optical images are shown in Figures S1–S3). Figure 1a presents a schematic of a typical three‐terminal back‐gated MoS_2_ field‐effect transistor used in our experiments. Figure 1b shows the transfer characteristics of the monolayer MoS_2_ FET at 1.8 K in linear and semi‐logarithmic scales. The monolayer nature was confirmed by photoluminescence (PL) measurements (Figure S4), which revealed a pronounced photoluminescence signal at 1.88 eV (660 nm) and 2 eV (618 nm) corresponding to the A_1_ and B_1_ direct excitonic transitions [38]. Such photoluminescence is absent in the bulk and few‐layer MoS_2_ sample [38]. The device exhibits n‐type conduction with a strong gate modulation, as expected for MoS_2_ FET [39]. The on/off current ratio of I_d_ is about 10^5^ and a field‐effect mobility of 2.8 cm^2^/Vs, comparable to the mobility of similarly fabricated back‐gated devices [40, 41]. The threshold voltage (V_th_) shifts positively with decreasing temperature (See the transfer characteristic at variable temperatures in Figure S5), which is consistent with previous reports on MoS_2_ [42, 43]. This shift in V_th_ can be attributed to the increase in Schottky barrier and gradual suppression of thermionic charge transport when the temperature decreases. As the system decreases in temperature, electrons occupy lower energy states, which effectively increases the Schottky barrier. To counteract the reduced thermal smearing, an increased gate voltage can be applied to achieve similar current injection. This balance between reduced thermal energy of electrons and required gate voltage to activate the channel results in the positive threshold voltage shift [44].

Figure 1c shows the MR measured on a monolayer MoS_2_ as a function of an (external) magnetic field (perpendicular to the plane of the device current) in different regimes: subthreshold regime (V_g_ –V_th_ <0 V), over‐threshold regime (V_g—_V_th_ > 0 V) and at the threshold regime (V_g_ –V_th_ = 0 V). The observed MR shows no sign of saturation even at 9 T. The maximum value of MR (686%) was measured in the subthreshold regime under a magnetic field of 9 T and at 1.8 K where a low current of a few tens of pico‐amperes flows through the transistor channel. Operating the device closer to the threshold regime (V_g_ –V_th_ = 0 V) causes a remarkable decrease in the MR. The MR continues to decrease as the transistor approaches the over‐threshold regime (V_g—_V_th_ > 0 V). Indeed, when the transistor reaches the ON state (V_g—_V_th_ > 0), a current of a few hundred nA flows through the channel. In this operating regime, the maximum MR achieved was limited to 11.4%.

This result confirms that our FET‐MoS_2_ devices are much more sensitive to magnetic fields when the device is operated in the sub‐threshold regime. Therefore, charge carrier density affects the MR. In the sub‐threshold regime, a carrier density of 10^11^ cm^−2^ is present, and the maximum MR is achieved. By gradually increasing the gate voltage and turning on the device, the charge carrier density reaches 10^12^–10^13^ cm^−2^. We suggest that the charge transport at low carrier density and interactions with defect states are more sensitive to magnetic fields than charge transport that occurs through the conduction band edge at higher carrier density. We provide a detailed discussion on the transport mechanism later. We have carried out a similar set of measurements on few‐layer and bulk MoS_2_ FET devices to study the effect of the change of electronic properties (Figures S6 and S7). In addition to the changes in the band structure, transistor characteristics such as V_th_ and the electron mobility are dependent on the number of layers. Table S1 reports the characteristic parameters—field‑effect mobility, I_ON_/I_OFF_ ratio, and threshold voltage V_th—_measured at room temperature for three representative devices based on monolayer, few‑layer, and bulk MoS_2_ used in this study. From these data, the extracted mobilities range from a few to several tens of cm^2^ V^−1^ s^−1^, depending on channel thickness, while the I_ON_/I_OFF_ ratio lies between 10^4^ and 10^6^. These values of µ, V_th_, and I_ON_/I_OFF_ are in good agreement with those reported for comparable MoS_2_ FETs on SiO_2_ substrates without high‑k dielectric encapsulation [45, 46].

**FIGURE 2:**
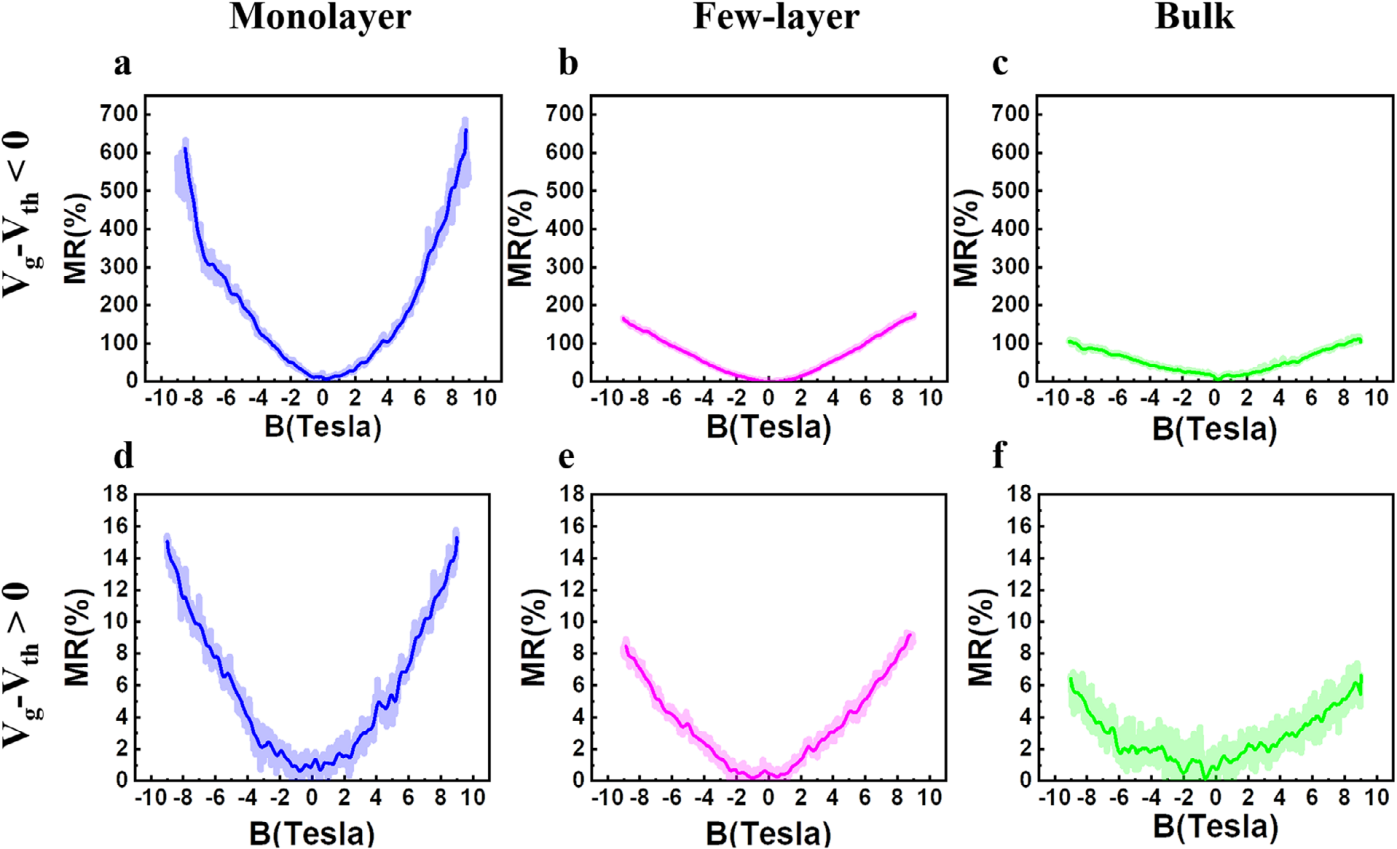
Gated magnetoresistance in gated devices following a change in MoS_2_ thickness: (a,b), (c) MR of monolayer, few‐layer, and bulk MoS_2_ in the subthreshold regime. (d,e,f) MR of monolayer, few‐layer, and bulk MoS_2_ in the on‐state. [measurement T = 1.8 K for all the plots shown in the present figure].

MR results at 1.8 K and in the subthreshold regime (V_g—_V_th_ <0 V) are shown in Figure 2a–c. The few‐layer and bulk devices also exhibit significant maximum MR exceeding 100% at 1.8 K. Maximum magnetoresistance values of around 176% and 116% were measured in few‐layer and bulk devices, respectively. However, compared with monolayer devices, these values are 4 to 5 times lower, which shows that monolayer MoS_2_ is the most sensitive to magnetic fields. Same MR measurements were done when the transistor is turned on, and the I_d_ current flowing through the channel is of the order of a few hundred nanoA (monolayer device) or a few microA (few‐layer and bulk devices) as shown in Figure 2d–f. A magnetoresistance is recorded even in the ON state of the transistors. A reduction of MR_max_ is observed with 15.7%, 9.2%, and 7.3% for monolayer, few‐layer, and bulk devices, respectively.

**FIGURE 3:**
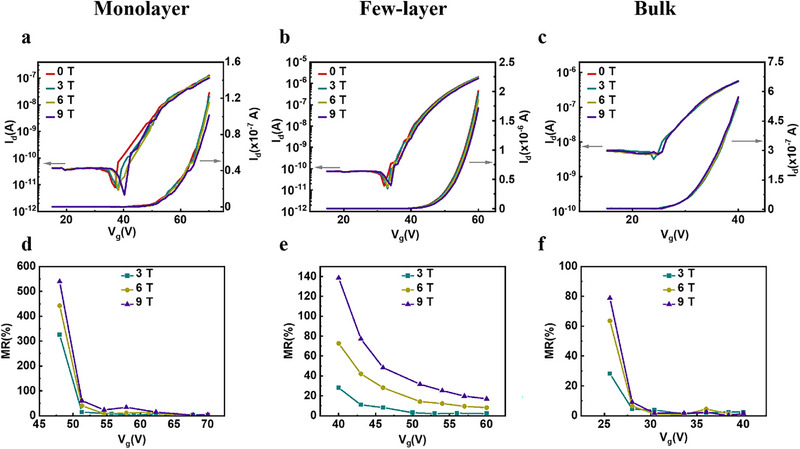
Effect of the magnetic field on the FET transfer characteristic of monolayer, few‐layer, and bulk MoS_2_. (a–c) Transfer characteristic curves measured at different magnetic field strengths. (d–f) Extracted MR as a function of gate voltage for monolayer, few‐layer, and bulk MoS_2_, respectively. [measurement T = 1.8 K for all the plots shown in the present figure].

Our MoS_2_ FET‐devices show a large sensitivity to an external magnetic field, which has not been observed in previous MoS_2_ reports [27, 47]. To reveal the origin of such gigantic MR, we characterized the gate‐induced current modulation as a function of magnetic field to understand the differences between the operating regimes (Figure 3a–c). The above‐threshold regime exhibits low dependence on the magnetic field. In contrast, the subthreshold regime of the transfer characteristic is strongly affected by the magnetic field, and a high MR is measured. Figure 3d–f shows the MR extracted from the transfer characteristics. A monotonic decrease of MR is observed for all the investigated material systems as the gate voltage increases, and the devices turn on. A similar trend is also observed at higher drain source voltages (Figure S8).

**FIGURE 4:**
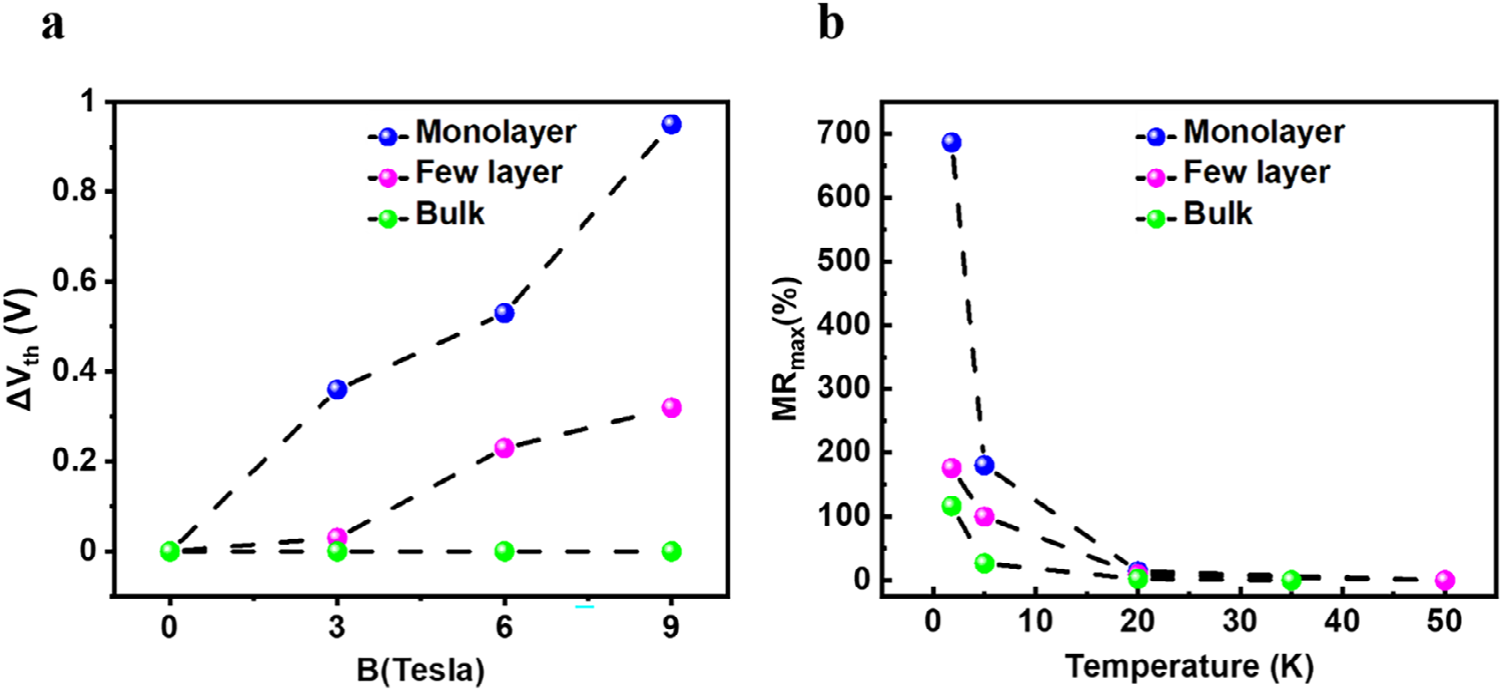
(a) Shift of the threshold voltage as a function of magnetic field at 1.8 K. (b) Evolution of the maximum magnetoresistance value (B = 9 T) measured for three FET devices having different channel thicknesses (monolayer, few‐layer, and bulk MoS_2_) as a function of temperature in the sub‐threshold regime.

We observe that the magnetic field causes a shift of the threshold voltage toward higher gate voltages in mono and few‐layer devices (Figure 4a). This shift of threshold voltage is highly temperature sensitive and is no longer detectable for T > 20 K, which explains the decrease in MR when the temperature is increased to 20 K (Figure S9). Indeed, the high MR values are exclusively observed below 20 K and are especially pronounced at 1.8 K within the sub‐threshold regime (Figure 4b). Next, we focus on the effect of the number of layers on the magnetoresistance in MoS_2_ crystals. To this end, we extracted the threshold voltage shift (ΔV_th_) as a function of magnetic field for the monolayer, few‐layer, and bulk devices at 1.8 K (Figure 4a). In the case of the bulk MoS_2_, we observe no shift in the threshold voltage, which is consistent with the smallest measured MR among the three systems. In few‐layer MoS_2_, we observe an almost linear dependence between the shift of the threshold voltage and the magnetic field, which becomes stronger in the case of monolayer MoS_2_. Our results show that the reduction of the number of layers turns the material more susceptible to magnetic fields, which causes the rapid increase of MR as a function of the number of layers. This dependency may originate from a convolution of multiple effects. The reduction of the bandgap with increasing layer numbers increases the number of free carriers, which is not beneficial, as demonstrated by the gate voltage dependency of the MR. In addition, the increased defect density in monolayer MoS_2_ compared to few‐layer and bulk devices may also contribute to the significantly higher MR, as it enables defect‐mediated transport mechanisms such as hopping and tunneling. Moreover, the spin‐valley locking, present in the monolayer, significantly suppresses scattering, which is not present in bulk material [48]. In the presence of a magnetic field, the spin‐valley locking is perturbed as evidenced by previous optical measurements that demonstrate a valley Zeeman splitting, which could cause an increase in the resistance only possible in monolayer MoS_2_ as the valley degeneracy is lifted [49]. In addition, we attribute the reduction in magnetoresistance to a change of dimensionality from purely 2D transport, where a perpendicular magnetic field confines carriers to orbits within the monolayer, to a quasi‐3D transport, in which interlayer coupling enables carrier motion across adjacent layers and disrupts the orbital trajectories through interlayer scattering, which reduces the effect of the magnetic field on the carriers.

**FIGURE 5:**
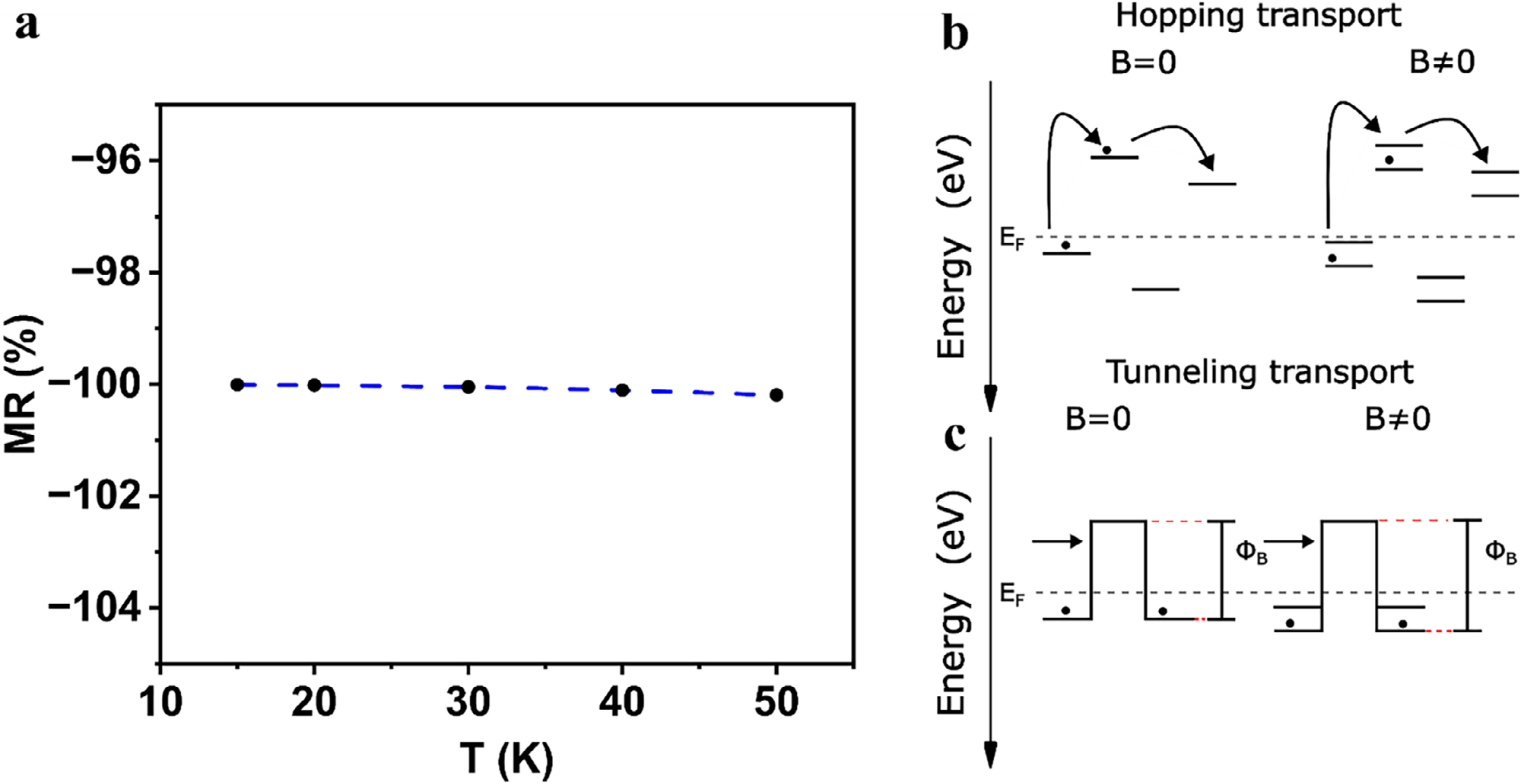
Simulated magnetoresistance and magneto transport mechanisms. (a) Simulated temperature evolution of magnetoresistance. (b) Schematic of the proposed mechanism of magneto transport by hopping transport. (c) Schematic of the proposed mechanism of magneto transport by tunneling transport. The tunneling barrier Φ_B_ increases in the presence of a magnetic field as a result of the Zeeman splitting of the defect states.

The increase of V_th_ in the presence of a magnetic field suggests a change in the energy levels of MoS_2,_ especially in the monolayer case [50]. Charge transport in MoS_2_ is generally considered to occur mainly through two mechanisms, which are thermally activated conduction and defect‐mediated electron hopping or tunneling. Band‐like transport is achieved by significantly increasing the charge density through the gate voltage in the above threshold regime, i.e., placing the Fermi level close to the conduction band edge (CBE) [50]. To shed light on this charge transport mechanism, we have combined electronic properties obtained from Density Functional Theory with the Boltzmann transport equation (BTE). Electron and phonon band structures are obtained using Quantum Espresso. Spin‐orbit coupling was included. The EPW code is used to compute the phonon‐limited carrier mobility in the presence of a small magnetic field [51, 52, 53]. Our simulations suggest that the Lorentz force causes a negative MR of 100% at 15 K, which decreases further toward higher temperatures (Figures 5a; S10). While negative MR has been reported in bilayer MoS_2_ as a result of lifting weak localization conditions, the magnitude is significantly lower and is caused by breaking of time reversal symmetry and not by Lorentz force [28]. Therefore, we conclude that the origin of the high MR lies in mechanisms beyond band transport.

An alternative conduction mechanism is hopping between mid‐gap states [54, 55, 56]. Defects such as sulfur vacancies are readily available in MoS_2_ monolayers with a density of 10^11^ cm^−2^ to 10^13^ cm^−2^. The states arising from these defects are positioned about 0.23–0.65 eV below the CBE [57]. At low temperatures (8 K – 100 K), band‐like transport is limited by the scarcity of electrons in the conduction band, which translates into a change of slope in the Arrhenius plot (Figure S11). Instead, charge transport is facilitated by electron hopping within energetically close mid‐gap states, e.g., Mott variable range hopping (VRH), Efros‐Shklovskii (E‐S) VRH [55].

Below 4 K, the hopping process is driven by Fowler‐Nordheim (F‐N) tunneling [22]. The transport mechanism can be selected through the choice of temperature and gate voltage, which are differently affected by the magnetic field. In the ON‐state, the devices exhibit low MR, and the FET mobility remains almost unaffected (1%), which suggests that the conduction band is not affected by the magnetic field. On the other hand, in the sub‐threshold regime, the devices show a high MR, indicating a strong effect of the magnetic field on the charge transport through mid‐gap states. In addition, the observed shift in threshold voltage shows that the energy alignment of the states changes with respect to the Fermi level when applying a magnetic field. These defect states are susceptible to magnetic fields. For example, the electronic states arising from sulfur vacancies are mainly localized on d‐orbitals of neighboring Mo atoms and slightly on p‐orbitals of adjacent S atoms [58]. Zeeman splitting of sulfur vacancies has been confirmed through magneto‐spectroscopy at 4.2 K [59]. The Zeeman splitting affects the variable range hopping charge transport by manipulating the defect energy levels with respect to the Fermi level as well as lifting the degeneracy of the states, which leads to the increased threshold voltage. The increased energy spread of the defect states causes a decrease in hopping probability, which hinders the charge transport (Figure 5b). Moreover, additional effects such as an increased localization of the wave function of the mid‐gap state may also contribute to the high MR as observed for WSe_2_ [22]. We propose that these effects underpin the mechanism that causes the increase in MR at 20 K compared to higher temperatures.

**FIGURE 6:**
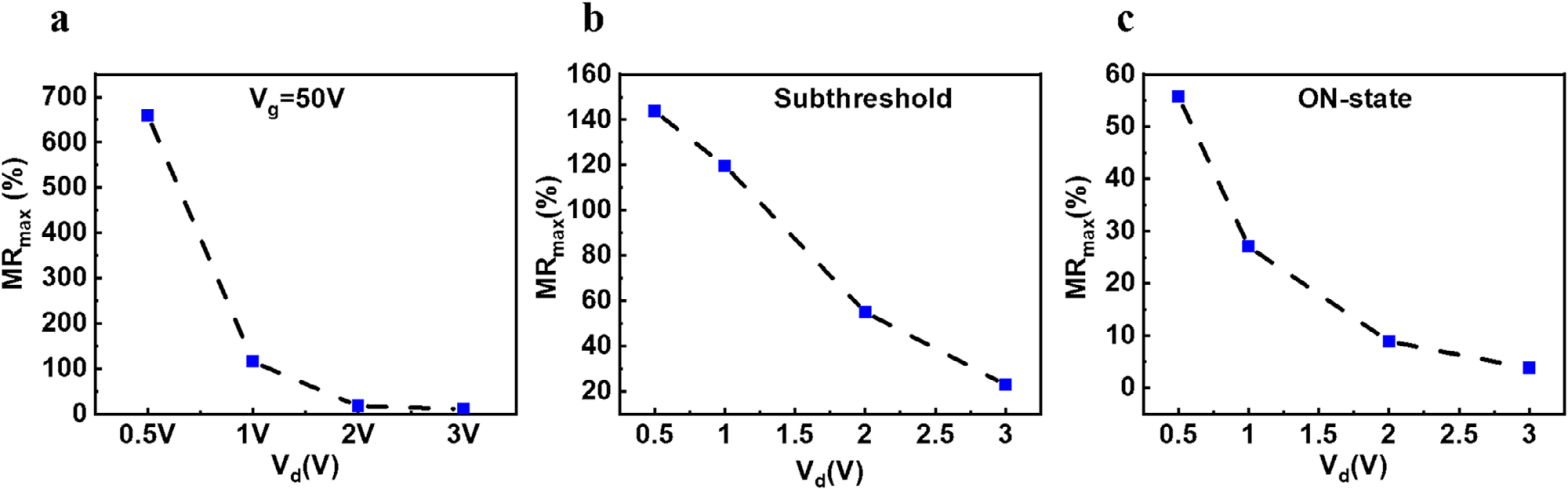
MR_max_ (at B = 9 T) evolution as a function of drain voltage at 1.8 K for the ML device in different carrier density conditions: (a) at V_g_ = 50 V, (b) in the subthreshold regime, and (c) in the ON‐state at V_g_ = 70 V. In (b), the gate voltage is adjusted to remain in the subthreshold regime as V_d_ increases.

Below 4 K, charge transport occurs through F‐N tunneling as demonstrated by the temperature independence of the conductivity [60]. In this regime, we observe an increase in MR from 25% at 20 K to 680% at 1.8K, which suggests a strong effect of the magnetic field on the tunneling process. The magnetic field causes the mid‐gap states to Zeeman split, which increases the energy distance between the mid‐gap state and the potential well, i.e., the tunneling barrier (Figure 5c). In addition to the magnetic field, the tunneling barrier can be tuned via the gate voltage, which explains the strong dependence of the MR on the carrier density. By gradually increasing the gate voltage, the Fermi level will enter the higher energy defect state, at which point the increase in tunneling barrier through Zeeman splitting vanishes, and a similar state as in the absence of the magnetic field is recovered. This effect is reflected by the similar current levels above the threshold regime, i.e. the vanishing MR as shown in Figure 3. When the device is turned on (at high gate voltage), the Fermi level approaches the potential well energy level, which prepares conditions in the channel for a low tunneling barrier. By reducing the gate voltage toward the subthreshold regime, the Fermi level is positioned between the Zeeman split states, and the tunneling barrier is increased, which turns the channel more resistant. Such a mechanism explains the strong and abrupt dependence on gate voltage as the conductivity decreases exponentially with the tunneling barrier. Furthermore, the F‐N tunneling current depends on the longitudinal electric field and therefore provides a tuning knob to manipulate the MR. We found that at a fixed gate voltage, the MR reduces drastically with increasing V_d_ (Figure 6a). However, the fixed gate voltage causes the device to operate in the subthreshold regime at low V_d_ and in the ON regime toward higher V_d_. To disentangle the effect of the carrier density on the MR, we traced the evolution of MR with drain source potential in the subthreshold regime and on‐regime by adjusting the gate voltage. In the subthreshold regime, we found a linearly decreasing MR (Figure 6b). In contrast, the MR follows a reciprocal relation with V_d_ in the ON‐state (Figure 6c). To analyze the mechanism governing the MR, we derived an expression for the MR assuming a F‐N tunneling current and a slight modulation of the tunnel barrier caused by the magnetic field (details in section 3). We found a good qualitative agreement between our model and the experimental data (Figure S13). We therefore conclude that the magnetotransport at 1.8 K is driven by F‐N tunneling and identify this mechanism as the most suitable to achieve high MR.

## 3 Conclusion

In this work, we demonstrate the tunability of the magnetoresistance through control of the charge carrier density in FET devices based on MoS_2_ layers, and identify device operation conditions to achieve high MR. We found that the subthreshold regime provides optimal conditions for high sensitivity with an MR of up to 680% at 1.8 K without any trace of saturation. In comparison, a very low magnetosensitivity is obtained when the FET device is turned in its ON state. In addition, MR was found to increase inversely with the number of layers, going from bulk down to monolayer. We rationalize the increased sensitivity through a noticeable linear shift in the threshold voltage in the presence of a magnetic field. The strength of such a shift decreases significantly above 10 K and upon increasing the number of layers. Using density functional theory combined with the BTE, we show that the origin of the high MR cannot be explained by simply reasoning in terms of the Lorentz force. Instead, it is the modulation of the F‐N tunneling probability that causes the high MR when the FET is turned into the subthreshold regime, and such evidence is experimentally supported by a strong temperature dependence of the MR. Moreover, low longitudinal electric fields were also found to increase the strength of the MR. Our work provides insights into the design and charge transport of 2D devices to achieve high MR for applications in magnetic sensing and memory.

## 4 Experimental Section/Methods

### 4.1 Sample Preparation

SiO_2_/Si‐p^++^ substrates with 285‐nm thick oxide were cleaned by sonication in acetone, isopropyl alcohol IPA solution, and rinsed with de‐ionized water (DI) and dried under nitrogen flow. Afterward, the substrates were heated on a hot plate at 110°C for 5 min to remove any residual moisture from the surface and treated with a UV ozone cleaner (UV/O_3_) for 20 min. The gel‐assisted mechanical exfoliation technique using both scotch tape and a gel film (Gel‐Pak) was employed to produce nanometer‐thick molybdenum disulfide flakes from bulk MoS_2_ crystals. These flakes were randomly transferred to the SiO_2_/Si substrate and mapped in terms of position, size, and relative thickness using an optical microscope (OM). Monolayer thickness was identified through photoluminescence measurements. The few‐layer thickness (5‐7 layers) is determined through optical contrast using OM. Then the substrates are spin‐coated with LOR (3A, Micro‐Chem) and positive photoresist of S1811G2 (Dow), and photolithography (laser writer: DWL 66Fs, Heidelberg Instruments) was employed to define the source and drain pattern. 50 nm thick gold Au with an adhesion layer (Cr) was deposited by using an e‐beam evaporator, followed by lift‐off processes.

### 4.2 Electrical and Magnetoresistance Measurements

All current‐voltage (I, V) electrical characterization and magnetoresistance measurements were carried out using a cryogenic system for characterizing charge, spin, and heat transport (PPMS Dynacool, Quantum Design Inc.) interfaced with a Keithley 2636 B sourcemeter. The PPMS system enables electrical characterization of FET‐MoS_2_ devices over a wide temperature range (from 1.8 to 400K) and in the presence of a magnetic field of up to 9 Tesla. All electrical and magnetoresistance measurements were carried out in the dark and under high vacuum (<10^−5^ Torr). Our three‐terminal devices, FET‐MoS_2,_ were stored in the Dynacool chamber under high vacuum for 12 h prior to measurements. Threshold voltages are extracted using the linear extrapolation method and defined as the intercept of the tangent at the point of maximum transconductance with the gate voltage axis in the transfer characteristic.

### 4.3 Density Functional Theory Calculations

The ground state electronic and phonon structure of the MoS_2_ structures were calculated within density functional theory and density functional perturbation theory, respectively, using the Quantum Espresso code [61, 62]. Spin‐orbit coupling terms were included using fully relativistic pseudopotentials from the pseudo‐dojo database [63]. The wave functions are expanded using a planewave basis set with a cut‐off energy of 95 Ry, and the Kohn‐Sham potentials are evaluated in Monkhorst‐Pack k‐ and q‐point grids of 12 × 12 × 1, and 9 × 9 × 1, respectively. The phonon‐limited transport properties were computed with the EPW code [51, 52]. To correctly account for long‐range electrostatic potential contributions to the electron‐phonon coupling [64], we included the effects of dynamical dipoles, but neglected the quadrupole effect, since its omission introduces a systematic error, irrelevant for the comparison of the systems under study. The EPW code is used to compute the phonon‐limited carrier mobility in the presence of a small magnetic field. This is achieved by first solving the BTE for the electronic occupation function, which accounts for the influence of both the electric field E and the magnetic field B. This equation incorporates scattering mechanisms through the total scattering lifetime, whose inverse is given by the scattering rate, involving electron‐phonon interactions computed in very dense grids. Once the occupation function is determined, the tensorial mobility can be calculated, which depends on the band velocity and the carrier concentration. From the field‐dependent mobility tensor, the magnetoresistance can then be derived by considering the changes in resistivity with and without the applied magnetic field. The Fermi level is positioned at the CBE to simulate electron (n‐type) transport as shown in the experiments. An external magnetic field of 0.1 T is applied to the system.

## Funding

Natural Sciences and Engineering Research Council (NSERC) of Canada. NSERC (funding Reference No. RGPIN‐2023‐ 0579), and the Fonds de recherche du Québec‐Nature et technologies (FRQNT). Ministry of Higher Education and Scientific Research of Tunisia for funding part of their PhD scholarship. CMC Microsystems MNT Awards. European Union's Horizon Europe research and innovation program under Grant Agreement No 101130384 (QUONDENSATE). “Materials for the Quantum Age” (QuMat) (registration number 024.005.006) is part of the Gravitation program financed by the Dutch Ministry of Education, Culture and Science (OCW). Sector Plan Program 2019–2023. This publication is part of the project “Quantum Materials by Design” with file number 2024.012 of the research program Computing Time on National Computer Facilities, which is (partly) financed by the Dutch Research Council (NWO) under the grant https://doi.org/10.61686/JUXDK41645. Support from the ERC SyG SKIN2DTRONICS (Contract No. 101167218) and the Italian Ministry of Education and Research (MIUR) in the framework of the FoReLab project (Departments of Excellence) is gratefully acknowledged.

## Conflicts of Interest

The authors declare no conflict of interest.

## Supporting information




**Supporting File**: smll72797‐sup‐0001‐SuppMat.pdf.

## Data Availability

The data that support the findings of this study are available from the corresponding author upon reasonable request.
