# Supplementary material for: Large Unsaturated Magnetoresistance in Gated MoS2 Flakes
Source: Small. 2026 Feb 11;22(21):e14561. doi: 10.1002/smll.202514561 (PMC13081104; doi:10.1002/smll.202514561)
Supplement: Supplementary file 1 — Supporting File: smll72797‐sup‐0001‐SuppMat.pdf. [file SMLL-22-e14561-s001.pdf]

Supporting Information

Large Unsaturated Magnetoresistance in Gated MoS_2 Flakes

*Anoir Hamdi,‡ Dominik Dettmann,‡ Andrés Rafael Botello-Méndez, Atiye Pezeshki, Lilian Skokan, Andreas Ruediger, Gianluca Fiori, Zeila Zanolli, and Emanuele Orgiu**

Table of Contents :

1. **Section 1: Optical and electrical characterization of MoS_2 devices**
   1.1. Optical microscope images of MoS_2 devices **(Figure S1, S2 and S3)**.
   1.2. PL spectra of monolayer MoS_2 (ML device) **(Figure S4)**.
   1.3. Transfer characteristics in linear and semi-logarithmic scales at different temperatures (from 1.8K to 300K) for the MoS_2 devices used in this work **(Figure S5, S6 and S7)**
2. **Section 2: Magneto transport in MoS_2 devices**
   2.1. Effect of the magnetic field on the FET transfer characteristic of monolayer, few-layer and bulk MoS_2 at V_d = 1V and extracted MR as a function of gate voltage **(Figure S8)**.
   2.2. Shift of the threshold voltage as a function of magnetic field at 5 K **(Figure S9)**
3. **Section 3: Arrhenius plot and magnetoresistance predictions from our simulation model**
   3.1. Temperature-dependent evolution of magnetoresistance (MR) as predicted by our simulation model **(Figure S10)**.
   3.2. Arrhenius plot of conductance in the 1L device for back gate voltages ranging from 10 to 70 V **(Figure S11 and S12)**.
4. **Section 4: Fowler-Nordheim tunnel equation and magnetoresistance**
   4.1. Derivation of an expression for magnetoresistance in the Fowler-Nordheim tunnel regime.
   4.2. Comparison of Fowler-Nordheim model and experimental magnetoresistance **(Figure S13)**.

Section 1: Optical and electrical characterization of MoS_2 devices

1.1 Optical microscope images of MoS_2 devices **(Figure S1, S2 and S3)**.

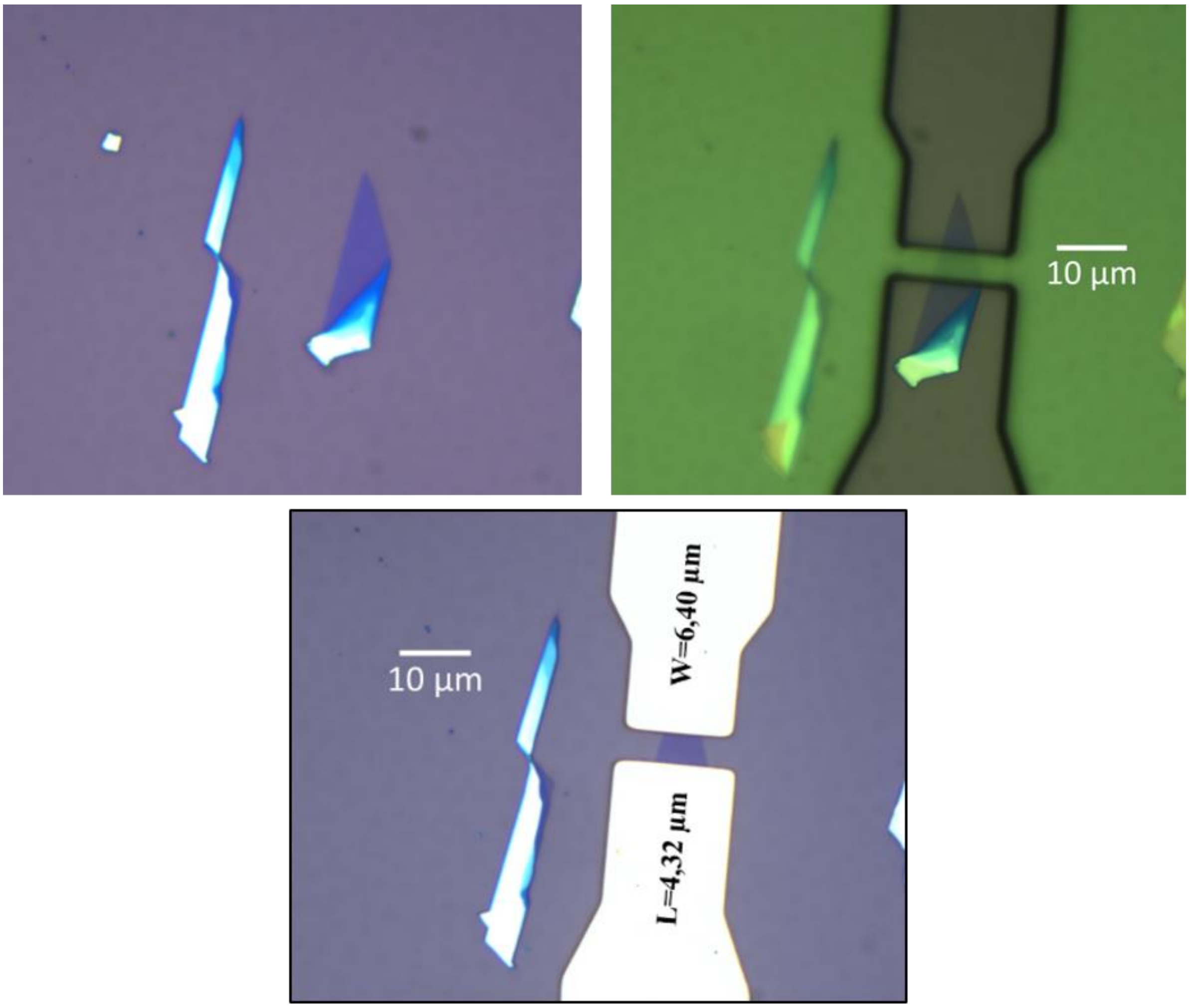

Figure S1. Optical microscope images of 1L-MoS_2 devices at different stages of fabrication: (a) after exfoliation; (b) after development; (c) after lift-off

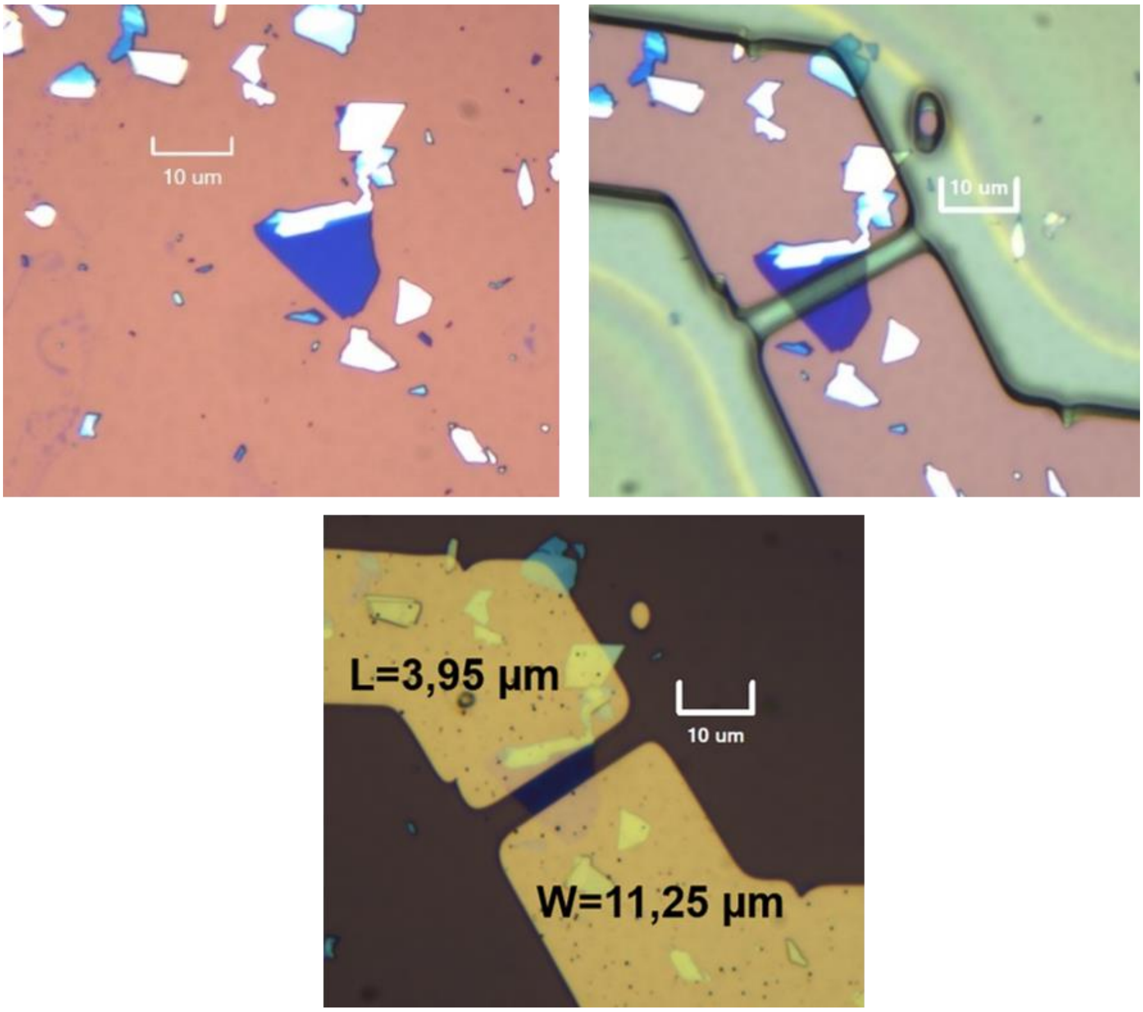

Figure S2. Optical microscope images of FL-MoS_2 devices at different stages of fabrication: (a) after exfoliation; (b) after development; (c) after lift-off.

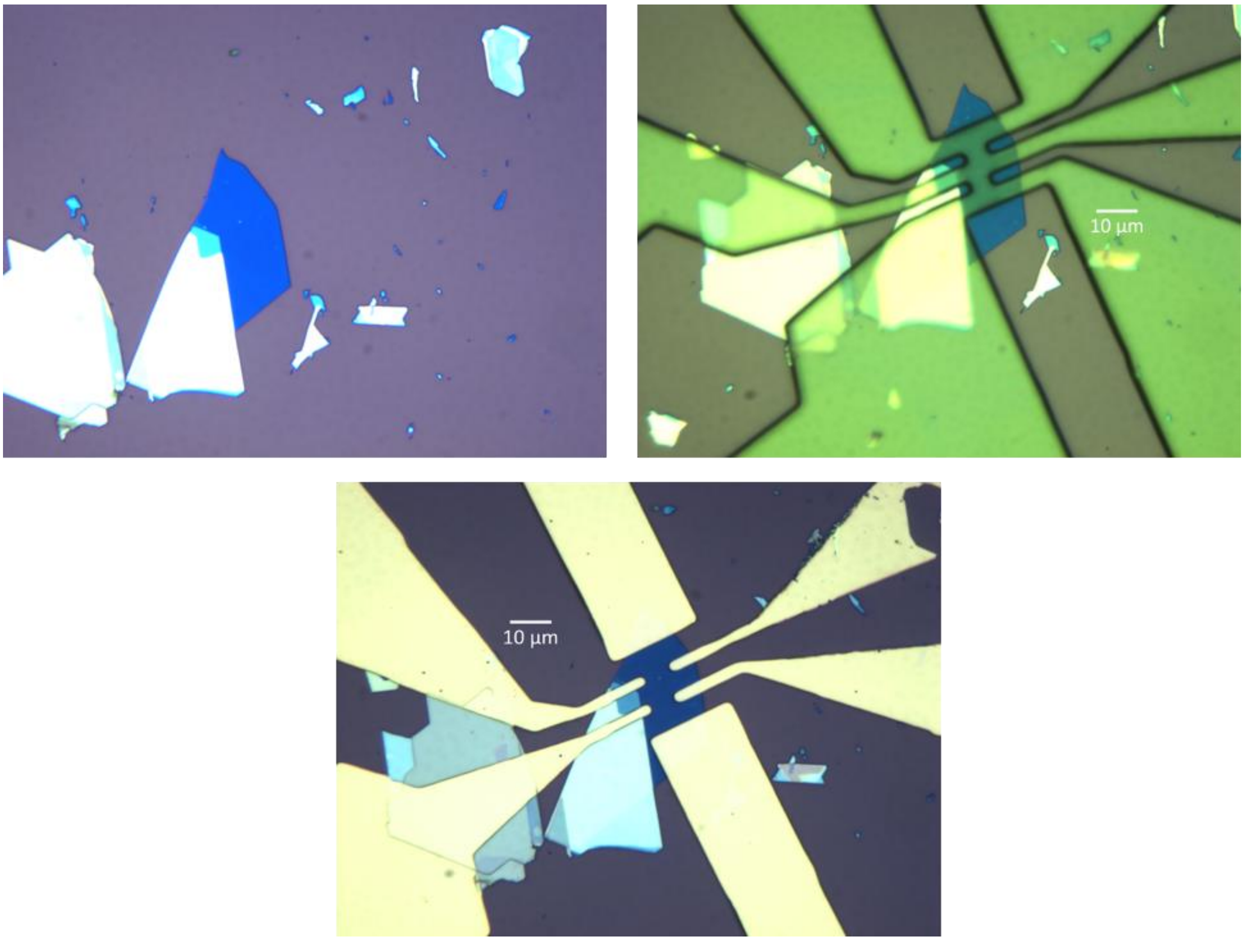

Figure S3. Optical microscope images of Bulk-MoS_2 devices at different stages of fabrication: (a) after exfoliation; (b) after development; (c) after lift-off

1.2. Photoluminescence spectra recorded on monolayer MoS_2 device.

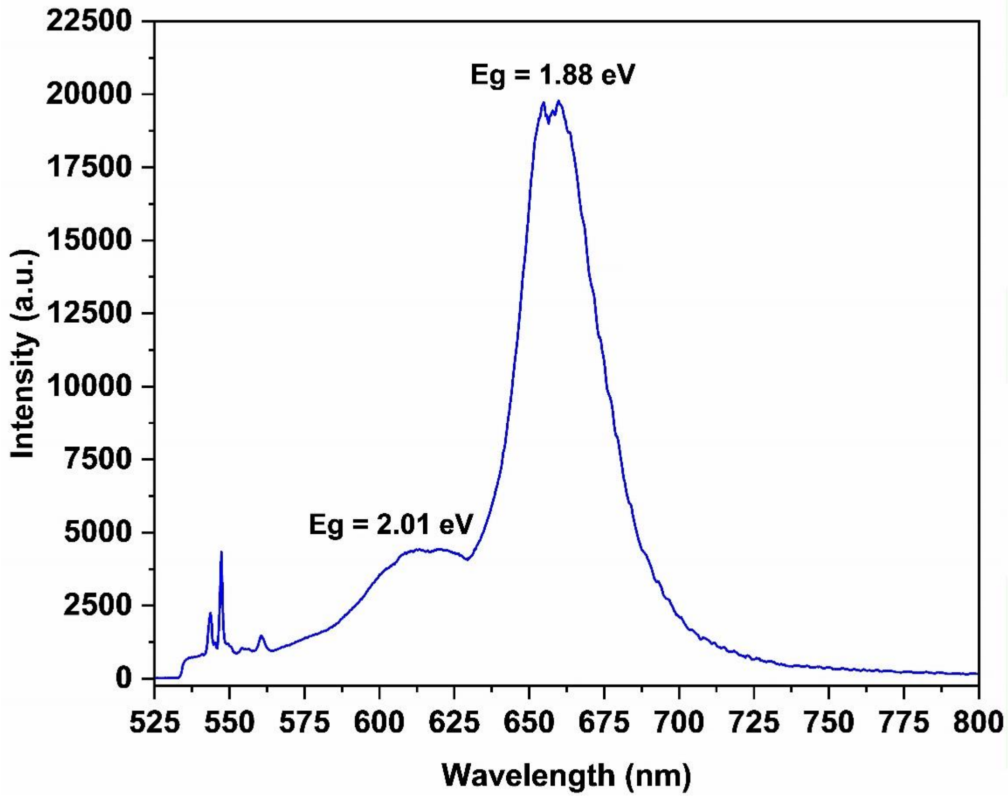

Figure S4. Photoluminescence spectra of monolayer (ML) MoS_2 device.

The monolayer nature of the MoS_2 was determined by photoluminescence (PL) measurements. A linearly polarized solid-state laser operating at 532 nm (Hübner Photonics, Cobolt Samba 25) was employed to excite the MoS_2 flake deposited on a Si/SiO_2 substrate. The excitation power was set to 54 µW. The laser beam was focused onto the sample using a 100× microscope objective with a numerical aperture (NA) of 0.7, achieving a spot size on the order of 1 μm^2. Backscattered light was collected in a confocal configuration and analyzed using a Tokyo Instruments spectrometer with a 500 mm focal length, equipped with a diffraction grating of 150 lines/mm. Spectral data were acquired over 100 seconds, yielding a spectral resolution of approximately 0.3 nm. Signal detection was carried out using a thermoelectrically cooled CCD camera (Andor iDUS 420 BUV) maintained at −70 °C to minimize dark current and enhance sensitivity. Figure S4 displays the photoluminescence spectrum corresponding to this device. Distinct luminescence emissions are observed at 1.88eV (660 nm) and 2.01 eV (618 nm), respectively corresponding to the A_1 and B_1 direct excitonic transitions [1]. Such luminescence is absent in bulk MoS_2 sample [1].

1.3. Transfer characteristics in linear and semi-logarithmic scales at different temperatures (from 1.8K to 300K) for the MoS_2 devices used in this work **(Figure S5, S6 and S7)**

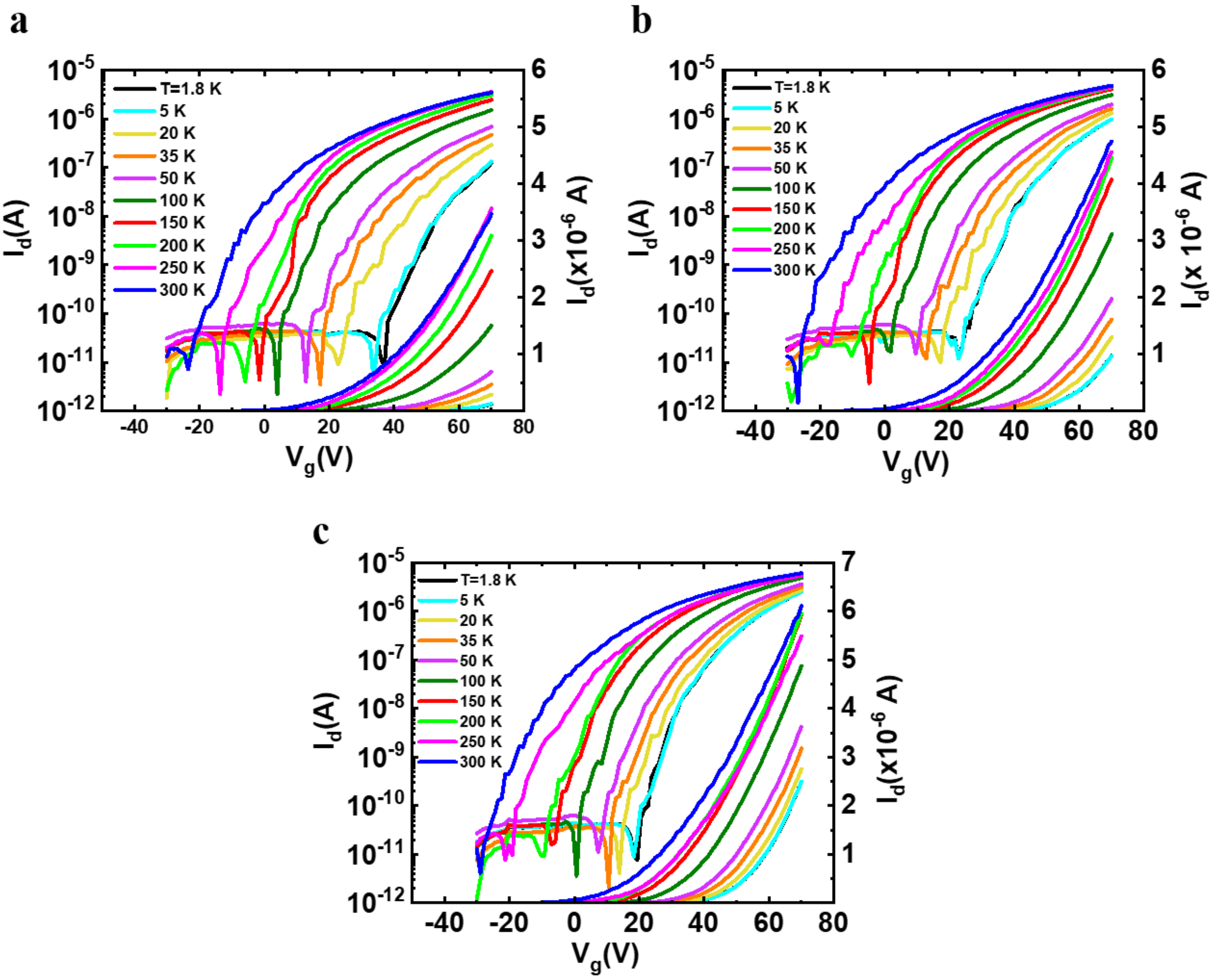

Figure S5. Transfer characteristics in linear and semi-logarithmic scales at different temperatures (from 1.8 K to 300 K) for the MoS_2 monolayer device, showing positive shifts in threshold voltage (V_{th}) with decreasing temperature: (a) Transfer characteristics at $V_d = 1$ V, (b) at $V_d = 2$ V and (c) at $V_d = 3$ V.

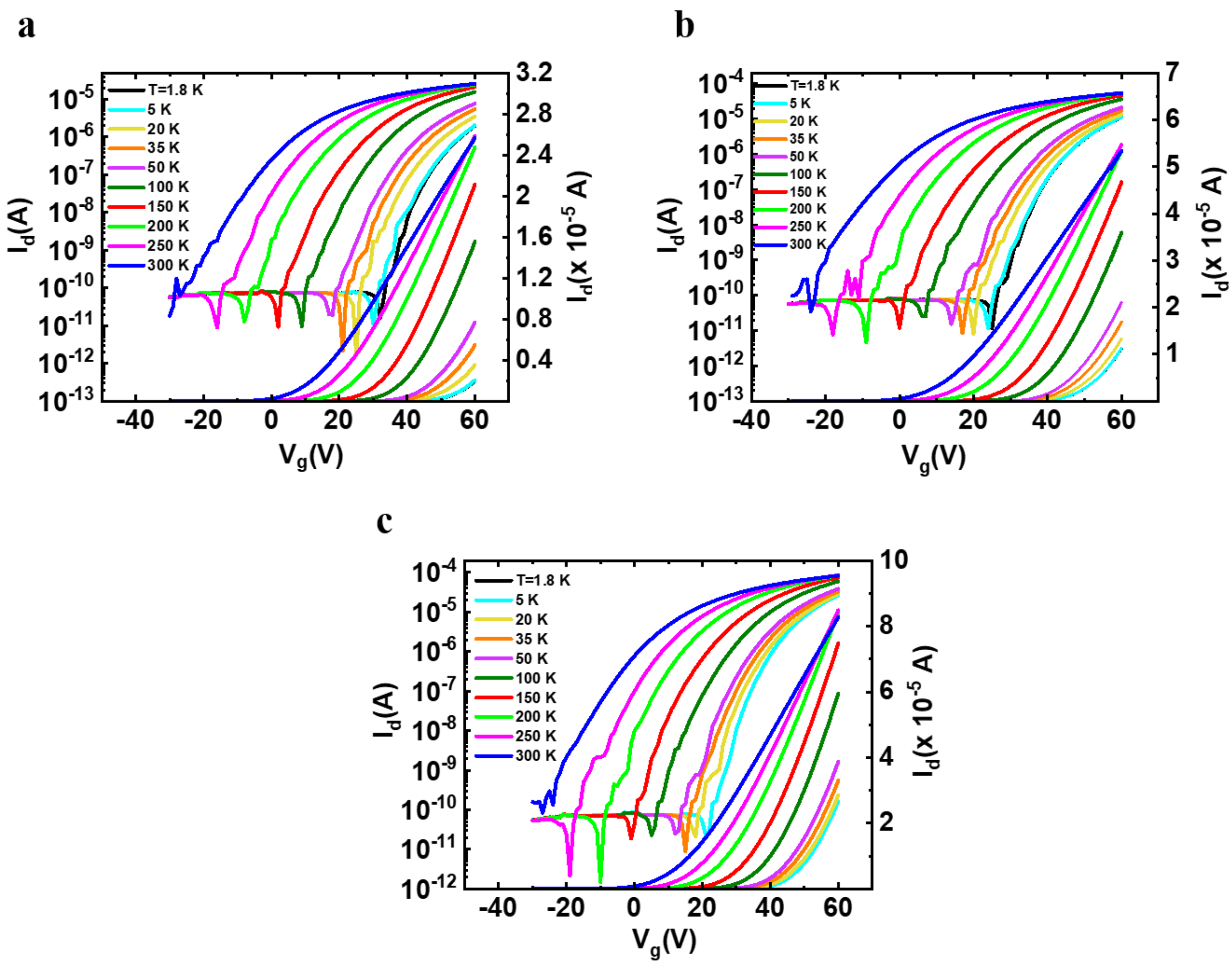

Figure S6. Transfer characteristics at different temperatures (from 1.8 K to 300 K) for the FL device at: (a) $V_d = 1$ V, (b) $V_d = 2$ V and (c) $V_d = 3$ V.

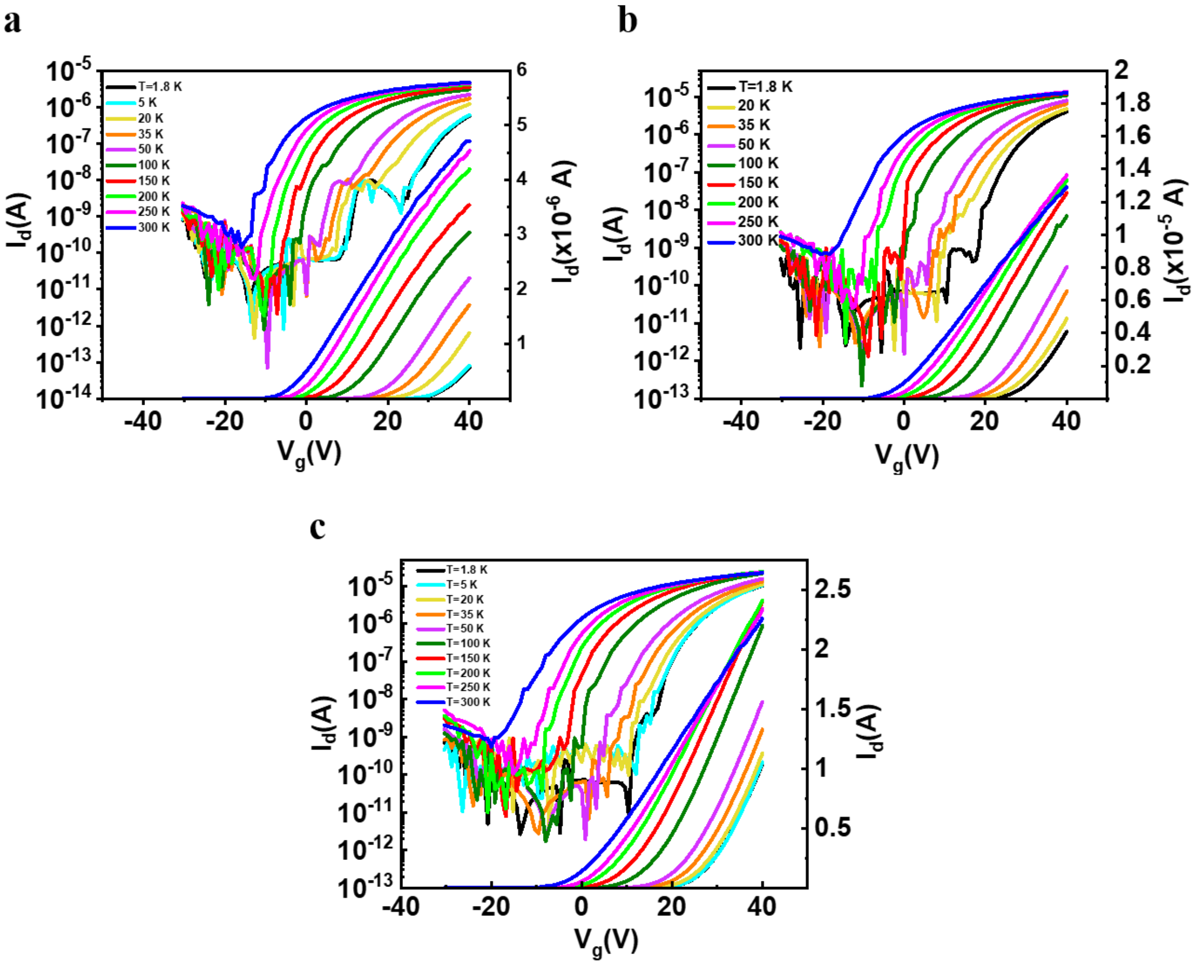

Figure S7. Transfer characteristics at different temperatures (from 1.8 K to 300 K) for the bulk MoS_2 device at: (a) $V_d = 1$ V, (b) $V_d = 2$ V and (c) $V_d = 3$ V.

Table S1. Summary of the extracted values of V_{th}, I_{ON}/I_{OFF} ratio, and the electron mobility determined for the MoS_2 FETs at room temperature.

**MoS_2**	**μ_e ($cm^2V^{-1}s^{-1}$)**	**V_{th} (V)**	**I_{ON}/I_{OFF}**
Monolayer	2.8	-2.7	~ $1x10^6$
Few-layer	17.3	-2.8	~ $1x10^6$
Bulk	4	-3.5	~ $5x10^4$

Section 2: Magnetotransport in MoS_2 devices

2.1 Effect of the magnetic field on the FET transfer characteristic of monolayer, few-layer and bulk MoS_2 at $V_d = 2$ V and extracted MR as a function of gate voltage **(Figure S8)**.

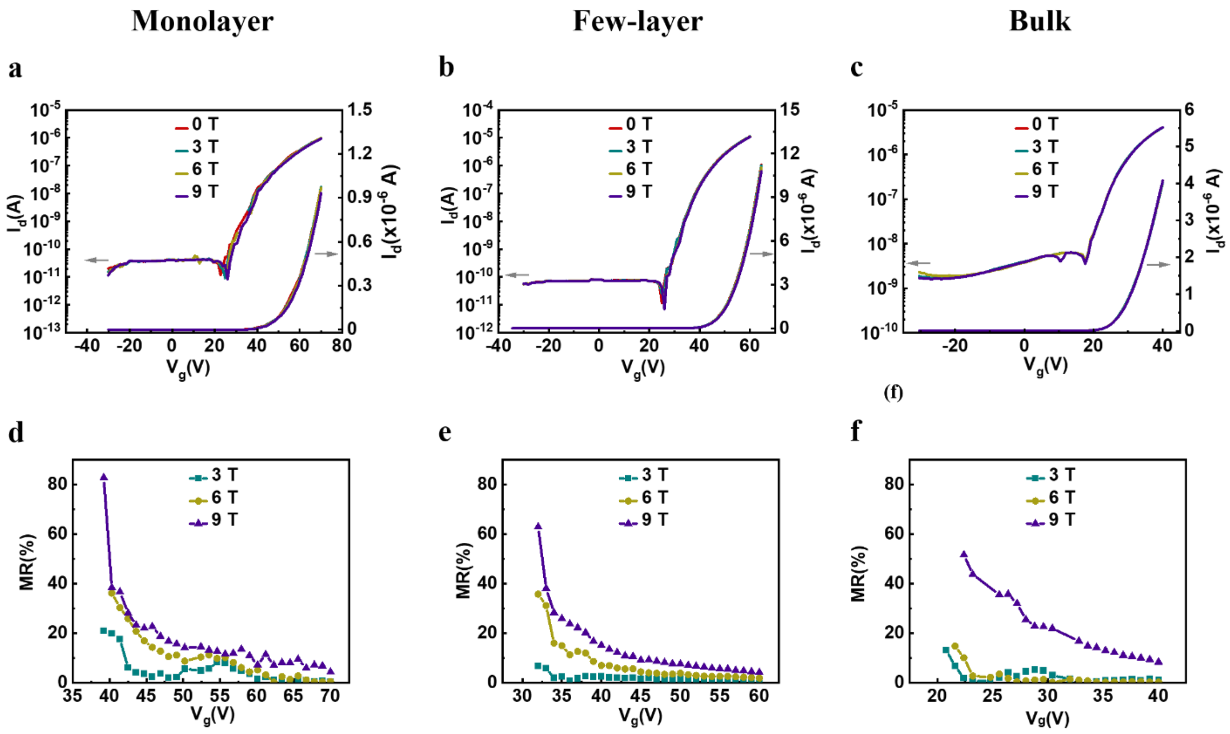

Figure S8. Effect of the magnetic field on the FET transfer characteristic of monolayer, few-layer and bulk MoS_2 at $V_d = 2$ V. a, b, c) Transfer characteristic measured at different magnetic field strengths. d,e,f) Extracted MR as a function of gate voltage for monolayer, few-layer and bulk MoS_2, respectively.

2.2 Shift of the threshold voltage as a function of magnetic field at 5 K **(Figure S9)**

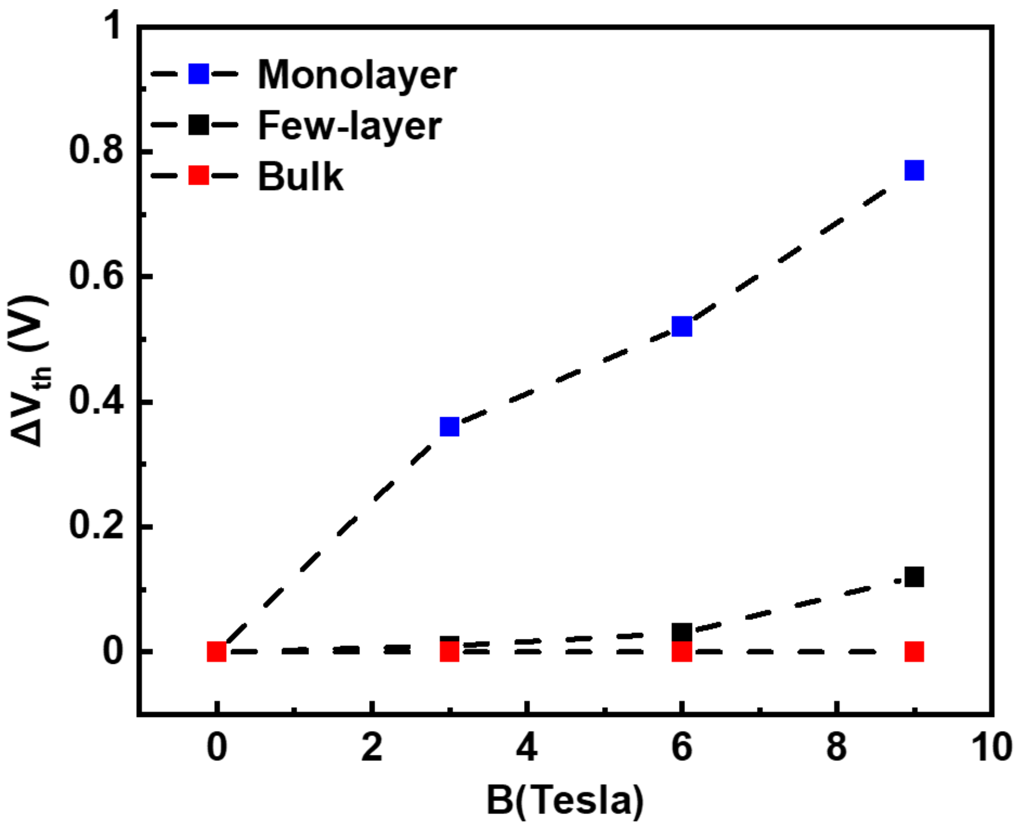

Figure S9. Shift of the threshold voltage as a function of magnetic field at 5 K.

Section 3: Arrhenius plot and magnetoresistance predictions from our simulation model

3.1 Temperature-dependent evolution of magnetoresistance (MR) as predicted by our simulation model **(Figure S10)**

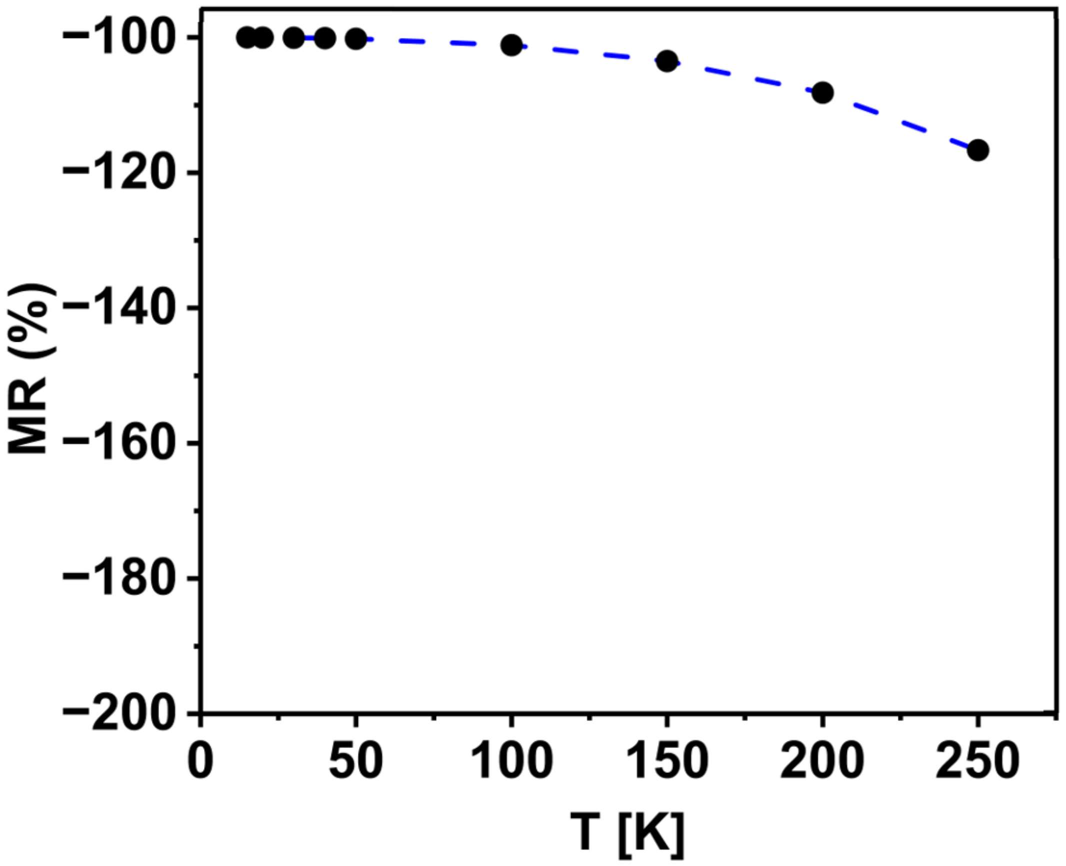

Figure S10. Simulated temperature evolution of magnetoresistance (MR) using electronic properties from density functional theory with the Boltzmann transport equation (BTE).

3.2 Arrhenius plot of conductance in the 1L device for back gate voltages ranging from 10 to 70 V **(Figure S11)**.

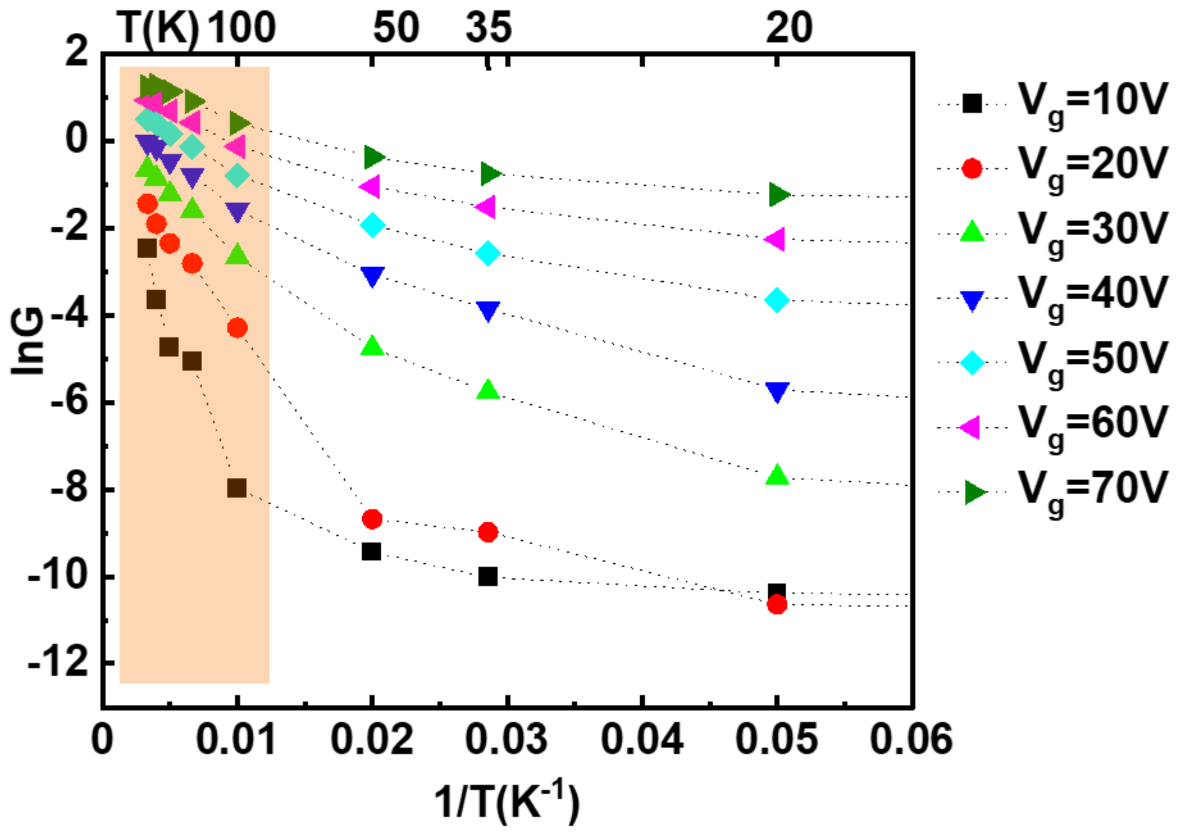

Figure S11. Arrhenius plot of conductance in the monolayer device for V_g ranging from 10 to 70 V. The region on the left marked by orange color is for the measurements at temperatures higher than 100 K, at which the transport in the MoS_2 flake is well described by the thermal activation mechanism.

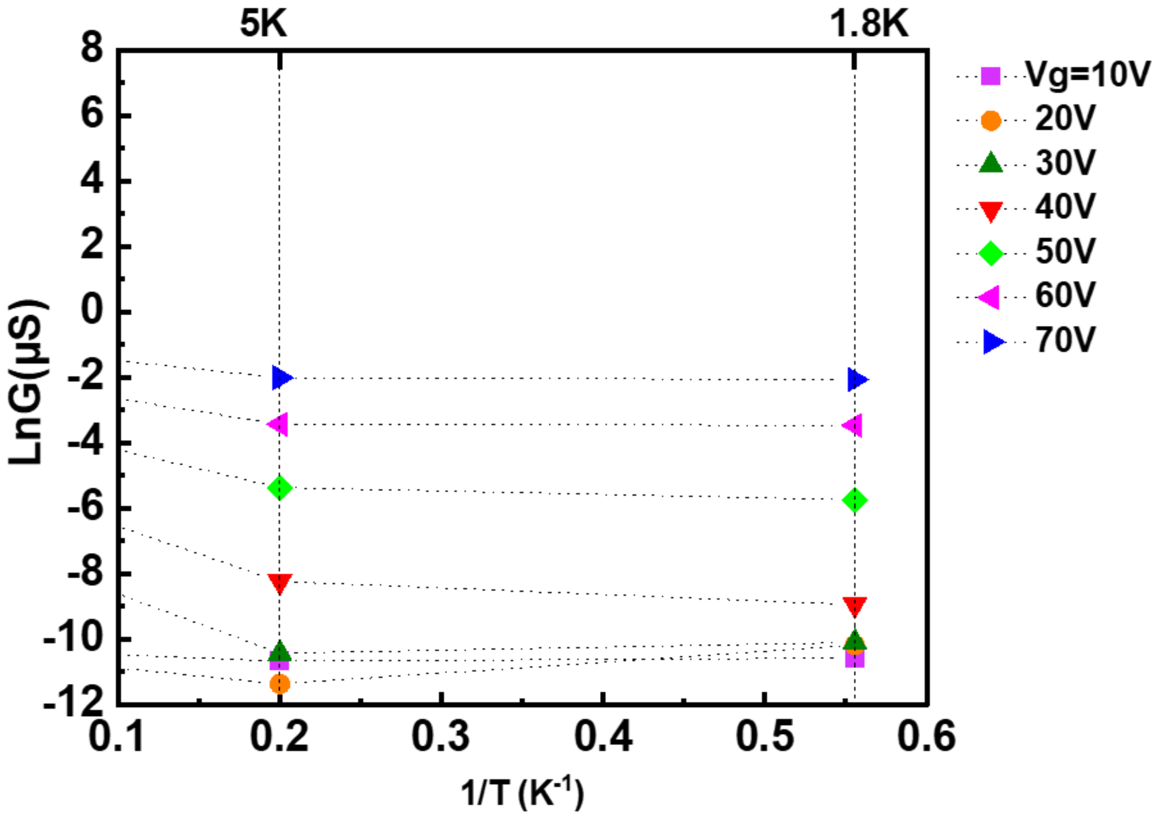

Figure S12. Arrhenius plot of conductance in the monolayer device for V_g ranging from 10 to 70 V, showing that conductivity remains temperature-independent for T < 5 K and confirming that charge transport in this regime occurs via Fowler–Nordheim tunneling.

Section 4: Fowler-Nordheim modelling of the magnetoresistance

4.1 Derivation of an expression for magnetoresistance in the Fowler-Nordheim tunnel regime.

We developed an expression for the magnetoresistance assuming charge transport occurs through Fowler Nordheim tunneling. The current is obtained from the following equation:

$$I = \frac{Aq^3 m^* d^2}{8\pi m h \phi} V_{DS}^2 \exp(-\frac{4d\sqrt{2m\phi^3}}{3\hbar q V_{DS}}) \tag{1.0}$$

Where A is the cross section, q is the elementary charge, $m^*$is the effective mass of the charge carrier, d is the distance of the tunnel region, ϕ is the tunnel barrier, V_{DS} is the drain source voltage and m is the effective mass within the tunnel region. The MR reads the following:

$$MR = \frac{R(B) - R(0)}{R(0)} = \frac{I(0) - I(B)}{I(B)} = \frac{I(0)}{I(B)} - 1 \tag{1.1}$$

After inserting 1.0 into 1.1, we obtain:

$$MR = \frac{\phi_B}{\phi} \exp\left(-\frac{4d\sqrt{2m}}{3\hbar q V_{DS}}\left[\sqrt{\phi^3} - \sqrt{\phi_B^3}\right]\right) - 1 \tag{1.2}$$

where ϕ_B is the tunnel barrier in the presence of an external magnetic field. We may assume that the change of the tunnel barrier follows a linear perturbation such that:

$$\phi_B = \alpha \cdot B + \phi \tag{1.3}$$

Inserting equation 1.3 in 1.2 yields:

$$MR = \frac{\alpha \cdot B + \phi}{\phi} \exp\left(-\frac{4d\sqrt{2m}}{3\hbar q V_{DS}}\left[\sqrt{\phi^3} - \sqrt{(\alpha \cdot B + \phi)^3}\right]\right) - 1 \tag{1.4}$$

We have used equation 1.4 to reproduce the experimental MR as a function of gate voltage (Figure S12). The model fits well our experimental data confirming that Fowler-Nordheim tunneling governs the charge transport.

4.2 Comparison of Fowler-Nordheim model and experimental magnetoresistance **(Figure S12)**.

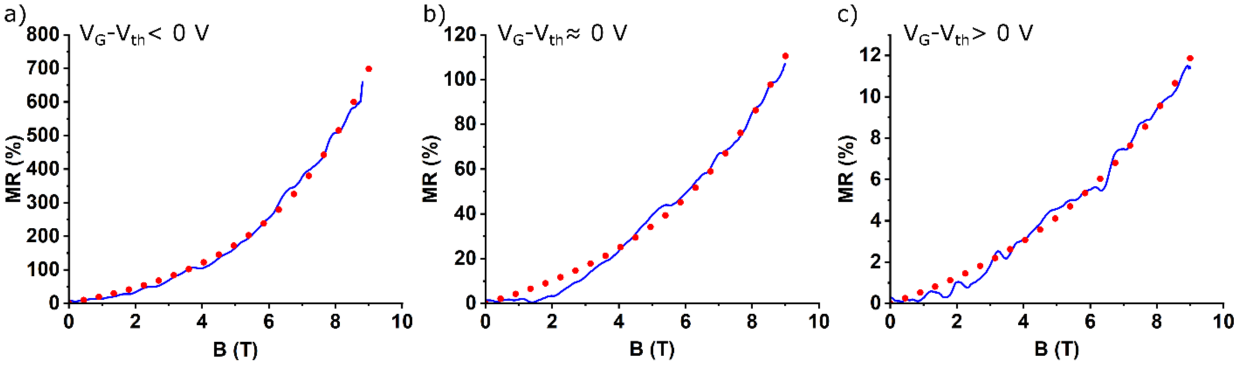

Figure S13. F-N modelling of the MR using the tunnel equation 1.4 for monolayer MoS_2. The experimental data (blue line) is well reproduced by the model (red dots) in the a) subthreshold, b) intermediate and c) ON-state at 1.8 K.

References

[1] Andrea Splendiani, Liang Sun, Yuanbo Zhang, Tianshu Li, Jonghwan Kim, Chi-Yung Chim, Giulia Galli, and Feng Wang. Emerging Photoluminescence in Monolayer MoS2. DOI: 10.1021/nl903868w. Nano Lett. 2010, 10, 1271–1275
